# Collagen matrices from sponge to nano: new perspectives for tissue engineering of skeletal muscle

**DOI:** 10.1186/1472-6750-9-34

**Published:** 2009-04-15

**Authors:** Justus P Beier, Dorothee Klumpp, Markus Rudisile, Roland Dersch, Joachim H Wendorff, Oliver Bleiziffer, Andreas Arkudas, Elias Polykandriotis, Raymund E Horch, Ulrich Kneser

**Affiliations:** 1 Department of Plastic and Hand Surgery, University Hospital of Erlangen, 91054 Erlangen, Germany; 2 Department of Chemistry, Philipps-Universität Marburg, 35032 Marburg, Germany

## Abstract

**Background:**

Tissue engineering of vascularised skeletal muscle is a promising method for the treatment of soft tissue defects in reconstructive surgery. In this study we explored the characteristics of novel collagen and fibrin matrices for skeletal muscle tissue engineering. We analyzed the characteristics of newly developed hybrid collagen-I-fibrin-gels and collagen nanofibers as well as collagen sponges and OPLA^®^-scaffolds. Collagen-fibrin gels were also tested with genipin as stabilizing substitute for aprotinin.

**Results:**

Whereas rapid lysis and contraction of pure collagen I- or fibrin-matrices have been great problems in the past, the latter could be overcome by combining both materials. Significant proliferation of cultivated myoblasts was detected in collagen-I-fibrin matrices and collagen nanofibers. Seeding cells on parallel orientated nanofibers resulted in strongly aligned myoblasts. In contrast, common collagen sponges and OPLA^®^-scaffolds showed less cell proliferation and in collagen sponges an increased apoptosis rate was evident. The application of genipin caused deleterious effects on primary myoblasts.

**Conclusion:**

Collagen I-fibrin mixtures as well as collagen nanofibers yield good proliferation rates and myogenic differentiation of primary rat myoblasts in vitro In addition, parallel orientated nanofibers enable the generation of aligned cell layers and therefore represent the most promising step towards successful engineering of skeletal muscle tissue.

## Background

Finding a suitable matrix has been a crucial step and still represents one of the main obstacles for tissue engineering of skeletal muscle [1-4]. Especially in the field of skeletal muscle tissue engineering there are great demands to be met in terms of biocompatibility, three-dimensional fabrication and above all the right balance of elasticity, stability and degradation. Several attempts have been pursued to test suitable materials with various consistencies, such as gels [5,6] , sponges [7] e.g. and most recently also electrospun nanofibers [8-12]. In this study our first aim was to develop and establish novel biocompatible matrices for myoblast cultivation. Furthermore, we intended to directly compare these novel matrices with varying different consistencies and micromorphologies to established tissue engineering matrices which had not yet been applied in skeletal muscle generation. Finally we wanted to assess and identify their advantages and disadvantages as matrices for skeletal muscle tissue engineering.

On one hand, collagen is a very promising material, especially for engineering of muscle tissue, since it mimics the natural extracellular matrix very closely and may thus contribute to myoblast proliferation and differentiation [13]. On the other hand pure collagen-gels show contraction of the matrix within 1–2 days [1]. To achieve more stability, we introduce a hybrid collagen-I-fibrin-gel in muscle tissue engineering research [14-16].

Biocompatibility in vivo is an essential demand on every matrix used for tissue engineering and therefore the application of materials such as Matrigel™ [17,18] or chicken embryo extract [19] was excluded in this study. In all our experiments we only used biocompatible materials which are also appropriate for clinical application. The use of fibrin-gels implicates the application of aprotinin as a fibrinolysis inhibitor. Aprotinin, however, has shown some allergenic potential in clinical trials [20,21]. We therefore tried to substitute aprotinin with genipin, which is an herbal substance that generates increased crosslinking of fibrin molecules and thereby delays degradation [22-24].

In this study a collagen-sponge containing a combination of several collagens was also evaluated for the purpose of skeletal muscle tissue engineering. Due to its irregular pore-size and high elasticity, it seemed to be a promising matrix and has only recently been introduced in skeletal muscle tissue engineering [7]. Additionally, we tested OPLA^® ^(open-cell poly-lactic-acid) a synthetic material, which was evaluated successfully for bone tissue engineering [25].

As the most promising potential matrix for skeletal muscle tissue engineering we developed electrospun collagen-I nanofibers. Particularly the possibility of processing parallel aligned fibers is a very promising property for engineering parallelly aligned muscle cells or even myotubes [26]. Therefore, pure collagen nanofibers with an average diameter of 550 nm were used for in vitro skeletal muscle tissue engineering. Hence collagen I was electrospun parallel aligned with nonwoven collagen I nanofibers as control. Proliferation, differentiation and apoptosis of primary expanded rat myoblasts on different 3D-matrices were evaluated. Furthermore, degradation and matrix stability as well as micromorphology of the different matrices were assessed in order to determine the most promising matrix for 3D engineering of skeletal muscle tissue.

## Results

### Cell proliferation, matrix stability and statistical evaluation

**Figure 1:**
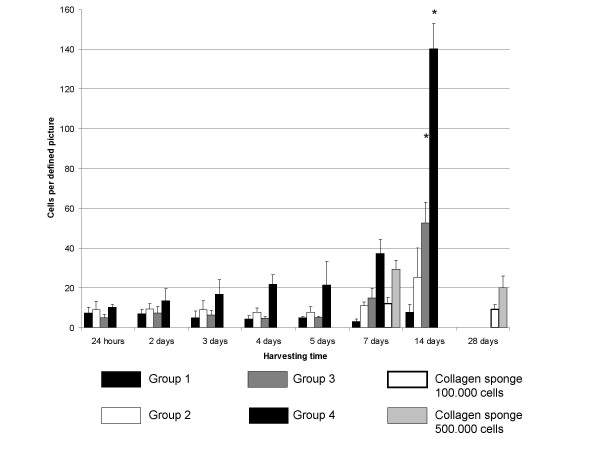
**Cell proliferation in collagen-I-fibrin gel and collagen sponges**. Group 3 and 4 (2,5 mg fibrin) showed satisfactory and significant (* p < 0,05) cell proliferation after two weeks. No significant cell proliferation could be detected in the collagen sponges.

Four groups of collagen-I-fibrin gels were evaluated (table 1): 5,0 mg/ml fibrin gels seeded with either 100.000 cells (group 1) or 500.000 cells (group 2) and gels with 2,5 mg/ml fibrin concentration also containing 100.000 cells (group 3) or 500.000 cells (group 4). In group 1 and 2, cell count showed only a slight increase of cell number over a period of 14 days. In contrast, the gels with a fibrin concentration of 2,5 mg/ml showed a significant increase of cell number (p < 0,05) after 14 days when seeded with 100.000 cells (figure 1). In gels with the same composition, but seeded with 500.000 cells, we could detect an even stronger increase in number of cells. The cell number did not increase (seeded with 100.000 cells) or was even decreased (500.000 cells) in collagen sponges.

**Table 1:** Evaluation of four groups of collagen-I-fibrin gels

Matrix		Seeded cells	Fibrin-concentration
Collagen I-fibrin gel	Group 1	100.000	5,0 mg/ml
	
	Group 2	500.000	5,0 mg/ml
	
	Group 3	100.000	2,5 mg/ml
	
	Group 4	500.000	2,5 mg/ml
	
Collagen sponge		100.000/500.000	

OPLA		100.000/500.000	

Collagen nanofibers 2D		20.000	

The stability of the collagen-I-fibrin matrices, as judged by the time of lysis of the matrix, showed a diametrically opposing behavior. The stability of fibrin-collagen gels was decreased with lower fibrin concentrations. However, in every group of fibrin-collagen gels the stability decreased over time until complete lysis occurred after day 21 (group 1 and 2), day 16 (group 3) or day 14 (group 4). Control scaffolds composed of 5,0 mg/ml or 2,5 mg/ml fibrin and 0,25 mg/ml collagen without cells clearly displayed higher stability (over 32 days for 5,0 mg/ml fibrin and 27 days for 2,5 mg/ml fibrin). There was a significantly higher proliferation of cells in the low fibrin groups (group 3 and 4) after 14 days as opposed to the other groups (groups 1 and 2) as assessed by one way ANOVA test with the Bonferroni correction for multiple comparisons.

The collagen sponges showed no shrinkage and no lysis within 4 weeks, so specimens were harvested after 4 weeks as the latest time point.

On the collagen nanofibers the cells adhered to the matrix as quickly as within 3–6 hours. The matrix was completely overgrown by cells after 10 days and cell proliferation, as measured by DAPI staining, was constant.

### Cell viability and differentiation

**Figure 2:**
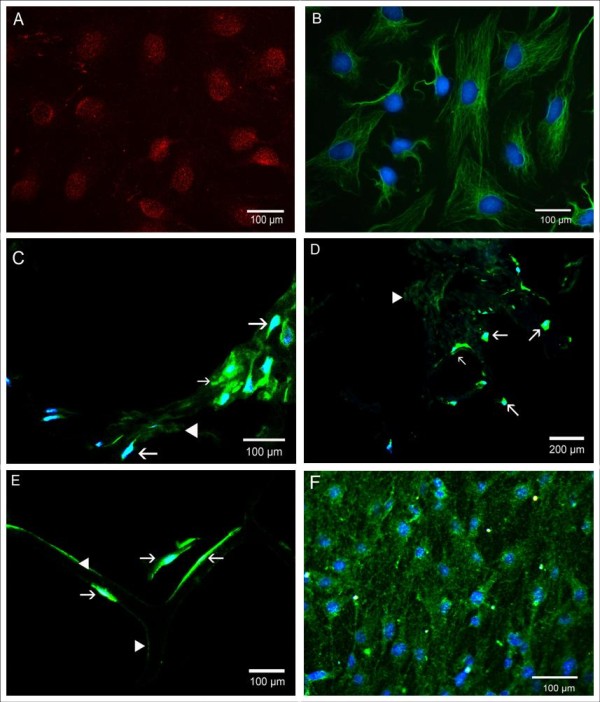
**Immunocytochemistry**. 2D-culture of myoblasts before implantation, 400× (A+B). A: for MyoD (red); B: for desmin (green); blue: nucleic counterstain with DAPI. C-E: myoblasts cultivated over 7 days in different matrices, immunostaining for desmin (green) and DAPI counterstain, arrows: desmin-positive myoblasts, arrow heads: unspecific fluorescence of the matrix. Ccollagen-I-fibrin gel, 400×. D: collagen sponge, 200×. E: OPLA, 400×. F: myoblasts after 24 hours on orientated collagen-I nanofibers, 400×.

**Figure 3:**
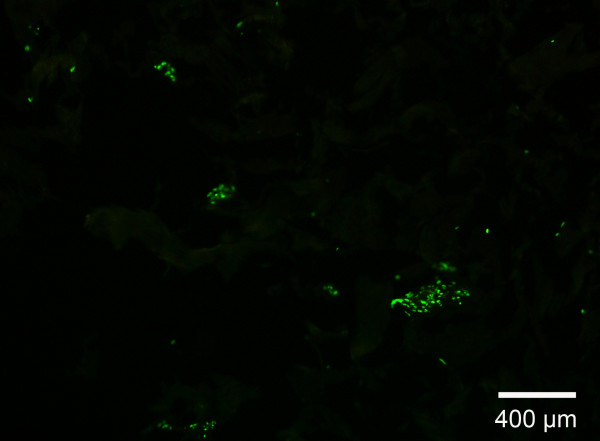
**TUNEL-assay**. Dead cells are stained with fluorescein (green), vital cells are stained with DAPI (blue), after 4 weeks of cultivation all cells are fluorescein-positive in the collagen sponge

**Figure 4:**
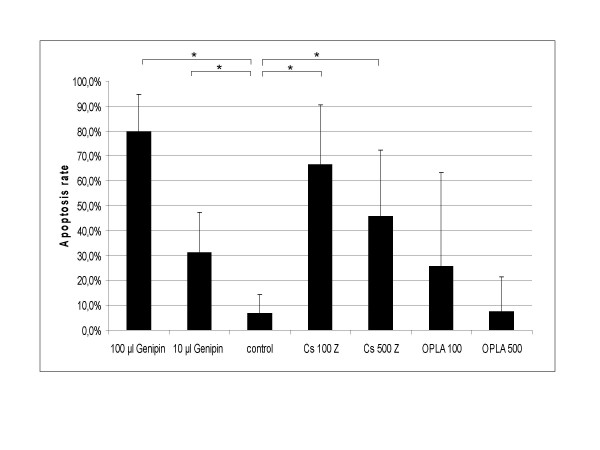
**Statistic analysis**. Apoptosis rate after four days in culture in collagen sponges (Cs) seeded with different cell concentrations and collagen-I-fibrin gel with different genipin concentrations (G) and control group containing aprotinin. Brackets indicate significant differences assessed by student's t-test (p < 0,05). Error bars: standard deviation of the mean.

In every matrix we could detect MyoD- and desmin-positive cells after 5 or 7 days, as determined by positive immunocytochemistry (figure 2). Apoptosis rates, assessed by TUNEL-assay (figure 3), ranged from 7% to 80%. The lowest apoptosis rate with only 7% was observed in the collagen-I fibrin gel containing aprotinin (group 4) which was used as a control. In gels with genipin-cross-linking, over 30% of the cells were apoptotic using 10 μl genipin. In gels containing 100 μl genipin (figure 4) apoptosis was significantly higher at 80%. No significant difference between peripheral sections (10 – 100 μm from the surface of the scaffold) and central sections was detected in the collagen-I fibrin gel groups.

Surprisingly, apoptosis rate in the collagen sponges were also high, ranging between 45% and 66% after four days in culture both significantly higher than the control group. The collagen sponges analyzed after 4 days in culture revealed a considerable difference between peripheral and central sections: The sponges seeded with 500.000 cells presented higher apoptosis rates in the middle, i.e. 54,7% (± 25,6 SDM) apoptotic cells in central sections versus 28,4% ± 19,9 SDM on the surface. In contrast to this expected result, the collagen sponges seeded with 100.000 cells revealed an increased apoptosis rate on the surface (76,4% ± 27,2 SDM on the surface versus 56,6% ± 18,2 SDM in the centre). Considering the high standard deviation of the mean, an interpretation of these findings is arguable. In contrast, OPLA^® ^showed low apoptosis rates between 26% and 8% and showed no significant difference to the control group.

TUNEL-assay of collagen-I nanofibers showed no apoptotic cells. All cells that were adherent to the matrix after four days in culture, were also DAPI-positive and fluorescein-negative. Due to the open structure of the nanofiber matrix non-adherent cells are washed out during medium exchange and washing steps before fixation. Thus non-adherent cells are not accounted for apoptosis analysis by the TUNEL-assay.

### Scanning electron microscopy (SEM) and phase contrast microscopy

**Figure 5:**
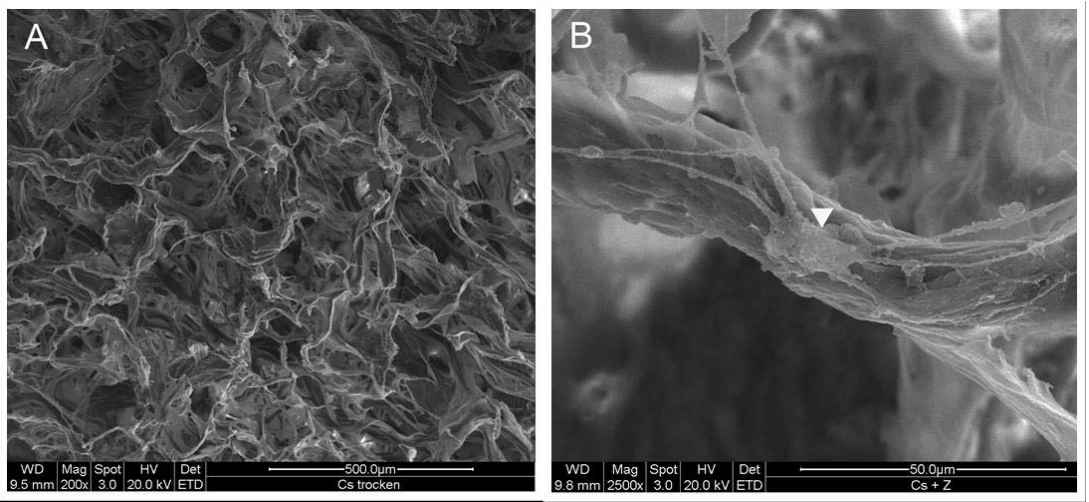
**SEM of the collagen sponges**. Surface of the dry sponge before cell seeding, 200× (A) After cell seeding: arrow-head: cell adhering to collagen fibrils, 2500× (A).

**Figure 6:**
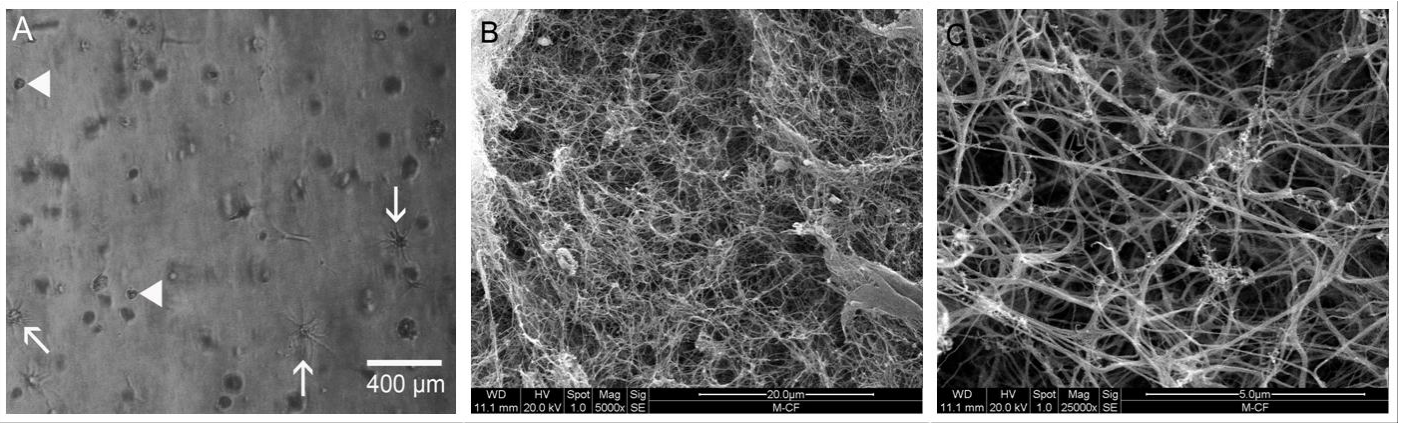
**A: phase contrast microscopy of collagen-I-fibrin gel:** 24 hours after seeding the cells (arrow-heads) and gel formation, some of the cells are already spread out (arrows), 100× B-C: SEM of the collagen-I fibrin gel after 10 days in culture media, 5000× (B) and 25.000× (C) after fixation for SEM and drying procedure the gel showed micro fibrils forming a sponge-like structure.

SEM-analysis of the collagen sponges showed an irregular structure with varying pore sizes (figure 5). After 7 days of culture the structure became more dense and clotted. Cells could be detected on the surface of the matrix, adhering directly to the matrix (figure 5b). Most cells were not evenly spread out like cells growing on collagen-I nanofibers but had a rather spheroidal shape. Phase contrast microscopy of collagen-I-fibrin gels confirmed that the cells were evenly spread over the whole matrix after the seeding procedure (figure 6). Surprisingly, the collagen-I-fibrin gel also showed micro fibrils forming a sponge-like structure in SEM analysis.

**Figure 7:**
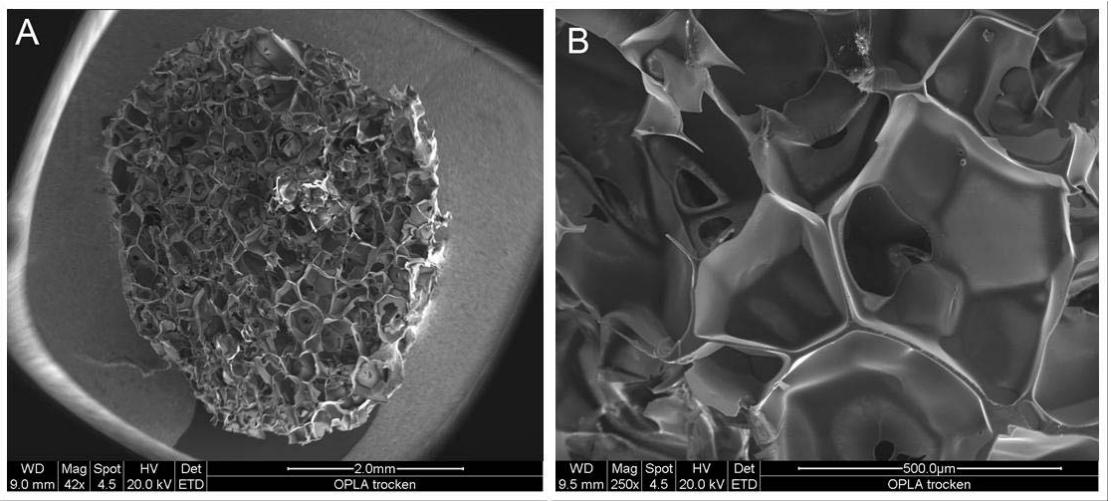
**SEM of dry OPLA-scaffolds (after in vitro culture OPLA-scaffolds dissolve during SEM-fixation)**. ****A**: 42×. **B**: 250×.**

Due to dissolution of OPLA^® ^in acetone, no SEM data could be taken after cell seeding and cultivation, but SEM-images of the dry matrix showed a rigid and sharply edged surface (figure 7).

**Figure 8:**
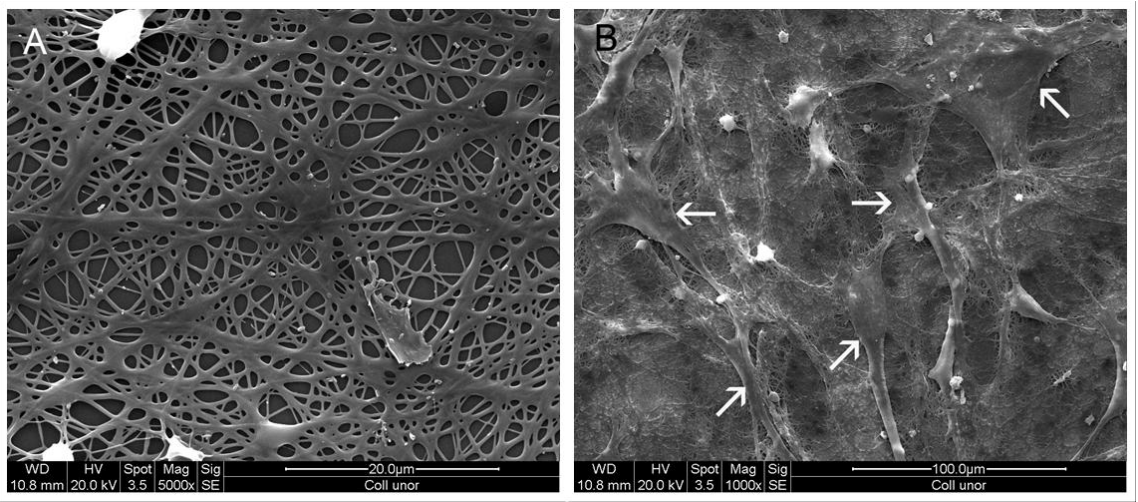
**SEM of unorientated collagen-I-nanofibers**. A: nanofibers without cells, the fibers show a more flattened structure with smaller pore sizes than orientated nanofibers, 5000×. B: nanofibers 3 days after seeding (arrows: cells), 1000×.

**Figure 9:**
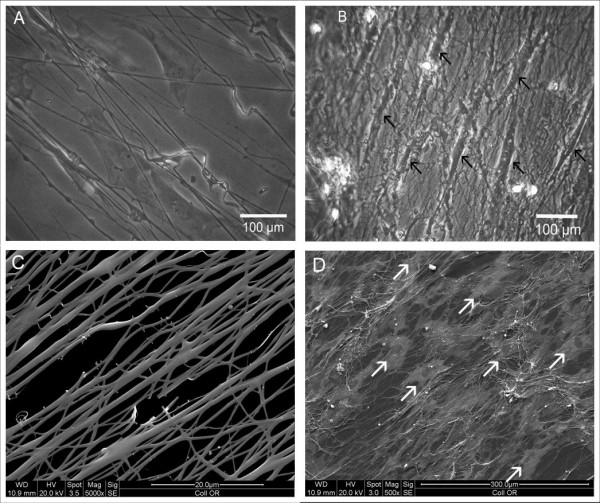
**Phase contrast microscopy, cells indicated by arrow-heads**. A: 24 hours after cell seeding most cells are already spread out, 300×. B: 7 days after cell seeding the cells grow in parallel alignment, 300×. SEMof orientated collagen-I-nanofibers after 3 days in culture. C: orientated collagen-I-nanofibers without cells, 5000×. D: parallel aligned cells on orientated nanofibers, 500×.

Compared to the orientated collagen-I nanofibers, SEM images of the nonwoven nanofibers showed a more flattened structure with smaller pore sizes (figure 8). Three days after seeding, the collagen-I nanofibers were completely overgrown with cells. A parallel alignment of myoblasts on orientated collagen-I nanofibres (figure 9) could be observed via phase contrast microscopy. This alignment was confirmed by scanning electron microscopy (figure 9c, d).

### Light-Cycler-PCR

**Figure 10:**
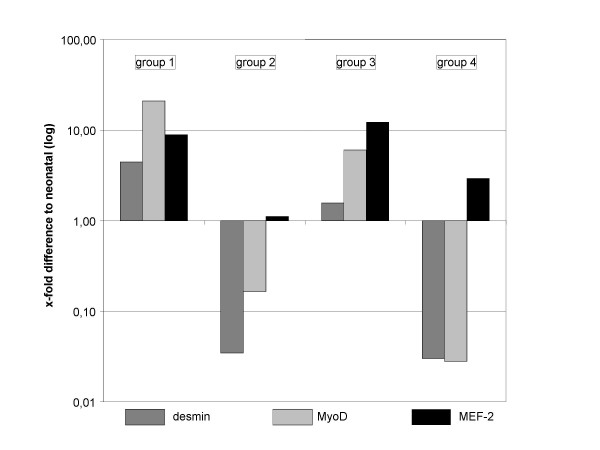
**Quantitative PCR: Expression of desmin, MyoD and MEF-2d in collagen-I-fibrin gels**. Group 1: desmin was 4-fold up-regulated (± 0,41 cycles), MyoD (21,11-fold ± 0,58) and MEF-2d (8,88-fold ± 0,37) were also clearly up-regulated in relation to neonatal muscle tissue. Group 3: over-expression of MyoD (6,06-fold ± 0,54) and MEF-2d (12,13-fold ± 0,98) but only slight up-regulation of desmin (1,57-fold ± 0,52). Group 2: desmin (0,03-fold ± 0,36) and MyoD (0,16-fold ± 0,29) down-regulated, MEF-2-expression (1,11-fold ± 0,05) was nearly equal to neonatal muscle tissue. Group 4: down-regulation of desmin (0,03-fold ± 0,09) and MyoD (0,03-fold ± 0,92), MEF-2d was nearly 3-fold over-expressed (2,93 ± 0,05).

After two weeks in culture (figure 10), desmin was 4-fold up-regulated (± 0,41 cycles) in group 1 of the collagen-I-fibrin gels and also MyoD (21,11-fold ± 0,58) and MEF-2d (8,88-fold ± 0,37) were clearly up-regulated in relation to neonatal muscle tissue. Group 3 also showed a distinct over-expression of MyoD (6,06-fold ± 0,54) and MEF-2d (12,13-fold ± 0,98) but only slight up-regulation of desmin (1,57-fold ± 0,52). In contrast, in group 2 desmin (0,03-fold ± 0,36) and MyoD (0,16-fold ± 0,29) were down-regulated whereas MEF-2-expression (1,11-fold ± 0,05) was nearly equal to neonatal muscle tissue. In group 4 desmin (0,03-fold ± 0,09) and MyoD (0,03-fold ± 0,92) were down-regulated but MEF-2d was nearly 3-fold over-expressed (2,93 ± 0,05) in relation to the calibrator. In collagen sponges the amount of extractable mRNA was too low and therefore excluded from analysis, the same observation was made in OPLA^® ^scaffolds. Collagen-I nanofibers developed in this study are two-dimensional, thus gene expression levels of myogenic mRNA was regarded as not comparable to those found in three-dimensional collagen-I-fibrin gel matrices.

## Discussion

Muscle tissue is frequently employed in plastic surgery for reconstructive purposes through transplantation of pedicled or free microvascular muscle flaps. Muscle transfer to treat muscle and soft tissue defects usually results in significant donor site morbidity. Tissue engineering of skeletal muscle could open new perspectives for reconstructive surgery by avoiding harvest of muscle tissue at the donor site. Therapy of congenital and degenerative muscle diseases could also benefit from findings in muscle tissue engineering research [27,28]. Natural components of the extracellular matrix like different collagen types seem to be suitable materials as matrix for culturing myoblasts. Furthermore, matrix components like fibrin or collagen have the advantage of clinical applicability and are therefore more attractive regarding regulatory issues compared to Matrigel™ matrix e.g. [29]. However, high contraction of pure collagen [30] or fibrin [5] gels has limited their application in vivo. Interestingly, the hybrid collagen-I-fibrin-gel analyzed in this study showed no contraction while significant cell proliferation and myoblast differentiation was evident. Expectedly, the parametric one way ANOVA-test showed significantly higher cell proliferation in the low-fibrin groups (group 3 and 4) after 14 days in culture than group 1 and 2 with twofold higher fibrin concentration. This is in accordance with the observations published by Cassell and Morrison et al [17] , who used pure fibrin gels with higher fibrin concentrations.

Surprisingly, quantitative PCR of muscle specific markers showed that expression rates did not correlate with different fibrin concentration rates but were rather associated with the number of cells seeded initially though this variable has been carefully controlled by using the same amount of total RNA. Desmin, MyoD and MEF-2d were clearly over-expressed in gels with low cell concentration (100.000 cells, group 1 and 3) but in contrast, high cell concentration (500.000 cells, group 2 and 4) provoked down-regulation of desmin and MyoD and slight up-regulation of MEF-2d in case of group 4. These findings suggest that muscle differentiation and gene expression also depend on cell-cell interactions and therefore the initial cell number may have a considerable influence on myoblast differentiation and tissue development. However, these results should be confirmed by further investigations. Additionaly, the use of GAPDH as endogenous control might be a limitation of the gene expression analysis. The variable of different fibrin concentrations is known to affect the expression rates of different genes. Just recently, Hong et al. demonstrated the influence of fibrin on the expression of collagen III and integrin ß3 in smooth muscle cells [31]. But at least to our knowledge varying fibrin concentrations in the range of 0,5 mg/ml versus 0,25 mg/ml does not effect GAPDH expression directly and is also not expected to influence the expression indirectly via parameters such as diffusion, gas exchange or mechanical properties of the matrix.

Unfortunately, the combination of collagen and fibrin gels did not provide enough stability required for in vivoapplication. In this context, crosslinking fibrin hydrogels with genipin appeared to be a good alternative to aprotinin to enhance the stability and possibly eliminate the allergenic risk at the same time. Unfortunately, our results showed increased apoptosis of the seeded cells depending on genipin concentrations, whereas in the control-samples (group 4) containing aprotinin a low apoptosis rate of 7% was found. A recent study proved a cumulative cytotoxicity of high concentrations of genipin (10 mM) in collagen gels [22]. Our results also confirm these findings for collagen-I-fibrin gels and for the lower concentration of 6 mM (10 μl genipin in the collagen-fibrin matrix).

In case of the tested collagen sponge, we did not detect significant cell proliferation even after four weeks in culture. The high density of the sponge after cultivation in medium could possibly be a reason for the high apoptosis rate of over 45% after 4 days in culture. Our results support the hypothesis that high density of a matrix may result in high apoptosis rates or low proliferation of myoblasts [32].

Analysis of OPLA^® ^proved to be difficult and remained limitative. Hence, no detailed conclusion regarding quantitative proliferation or gene expression could be drawn. After all OPLA^® ^as a synthetic material showed no particular advantage for muscle tissue engineering. The material may be well adapted for tissue engineering of bone tissue due to its inflexibility, but for muscle tissue engineering elasticity of OPLA^® ^is not sufficient.

In the past collagen has been electrospun with additives like PEO or elastin but these additives resulted in thinning (PEO) or breakage (elastin) of the fibers [8]. The nanofiber matrix we tested in this study was therefore composed of pure collagen I and showed a satisfying cell proliferation and maintenance of myogenic phenotype on both, orientated and unorientated nanofibers. The rapid adherence of the cells indicates a sufficient biocompatibility of the material. Additionally, orientation of the fibers seemed to provoke parallel aligned cell proliferation on every sample. The myoblasts were observed to orientate throughout the whole matrix. This phenomenon was described by various studies and can be explained as consequence of contact guidance. The contact guidance theory was well defined by Curtis et al [33] as reaction of the growing cell to discontinuities of the underlying substratum. Thus, the direction of developing pseudopodia and therefore cell growth can be directed by highly orientated matrices. Although Huber et al. [26] obtained orientated myotubes also on nylon 6/6 nanofibers, this synthetic material is not biodegradable and therefore unsuitable for clinical application. Furthermore, pre-coating of the nylon fibers with Matrigel^® ^was necessary to enable cell attachment. A similar approach was made by Riboldi et al, using highly polyesterurethane microfibres (DegraPol^®^) with Matrigel^® ^coating for their experiments [34]. They could also achieve myotube formation but in contrast to nylon 6/6, polyesterurethane is biodegradable. Unfortunately, the Matrigel^® ^coating is necessary for DegraPol^® ^also to enhance cell attachment, which represents the limitation of the work. This difficulty is avoided by electrospinning collagen-I nanofibers which combine biodegradability with rapid cell attachment and resembles the natural ECM particularly better than the afore mentioned materials.

### Limitations of this work

The comparison of entirely different matrices such as gels, nanofibers and sponges has to encounter the difficulty that the physical properties of the matrices like stiffness/elasticity, stability, diffusion of nutrients, pore size and many others differ in a wide range. Materials like collagen sponges and OPLA^® ^are already well characterized, hence the physical and chemical characterization has been limited to a minimum. Also fibrin and collagen gels as well as electrospun collagen I-nanofibres have been described in further details in previous studies. As a new approach this study tries to evaluate potential matrices with entire different structures for their practicability for muscle tissue engineering in a realistic clinical setting. But leaving out Matrigel coating or avoiding the use of cell lines as well as high-passage primary cells are great challenges and under such strict conditions the most basic demands on a possible matrix, i.e. sufficient stability, low apoptosis rates and high proliferation, cannot be met by various materials, which were successfully tested before under more academic conditions. Additionally, differentiation and even maintenance of myogenic phenotype of primary cells isolated from adult muscle tissue is a greater challenge than culturing cell lines like C2C12 or L6 which are used in a great number of studies. We therefore concentrated on the basic parameters such as proliferation rate, apoptosis and maintenance of myogenic phenotype rather than on further differentiation e.g. myotubes formation.

## Conclusion

Though collagen is known to be a suitable material for tissue engineering of skeletal muscle due to its resemblance to the natural extracellular matrix, our results confirm that the consistency of a matrix has a great influence on cell proliferation and differentiation. Despite its good biocompatibility the analyzed collagen sponge and also the OPLA^®^-scaffolds seem to be unsuitable for myoblast cultivation. In the collagen-I-fibrin gel high proliferation rates were yielded, while concomitant degradation of the matrix was observed. Further enhancement of the stability of the collagen-I-fibrin gel could result in a promising matrix for amorphic tissues.

Though two dimensional and still in its early stages, electrospun pure collagen-I nanofibers offer the unique property of parallel alignment of the cells in accordance to the contact guidance phenomenon. As the parallel orientation of differentiated myoblasts or myotubes, respectively, is one of the most important properties of a suitable matrix, we conclude that orientated nanofibres composed of collagen-I may be the most promising matrix for muscle tissue engineering at the moment.

## Materials and methods

### Cell isolation and culture

Primary rat myoblasts were isolated as previously described [35]. Briefly, the hind limbs' skeletal muscle of adult Lewis-rats was minced and digested first in 0,1% collagenase solution (Biochrom AG, Berlin, Germany) for 1 h and then with trypsin-EDTA (PAA Laboratories GmbH, Pasching, Austria) for 30 minutes at 37°C. After digestion the solution was filtered through cell strainers (Falcon, BD Biosciences, Bedford, MA, USA) with 70 μm pore size. The flow-through was centrifuged and the pellet was resuspended in DMEM (Dulbecco's Modified Eagle Medium Gibco/Invitrogen, Auckland, NZ) containing 10% FBS (fetal bovine serum) and 1% P/S (Penicillin/Streptomycin, Biochrom AG, Berlin, Germany), referred to as growth medium. The cells were then seeded in 75 cm^2^-culture flasks (Corning Incorporated, NY, USA), which had been coated with 10 mg/ml gelatin solution (Biochrom AG, Berlin, Germany) prior to seeding. The medium was changed every second day. For all experiments, myoblasts after the second passage were used. The ratio of myogenic cells in P_1_-cultures was determined by immunostaining for MyoD and desmin as described later (figure 2).

### 3D culture in collagen-I-fibrin-gels

Cells were detached from culture plates with trypsin-EDTA and centrifuged at 2000 rpm. After discarding the supernatant, the cells were resuspended in growth medium. Fibrinogen (Tisseel VH S/D kit, Baxter AG, Vienna, Austria) was dissolved according to the manufacturer's instructions and was used at a concentration of either 20 mg/dl (group 1 and 2) or 10 mg/dl (group 3 and 4). The fibrinogen solution was then mixed 1:1 with either 100.000 (group 1 and3) or 500.000 cells (group 2 and 4), aprotinin (3000 KU/ml,) and growth medium.

The collagen solution as provided in 0.02 N acetic acid (rat tail collagen type I, BD Biosciences, Bedford, MA, USA) was subsequently diluted with sterile water (aqua ad injectabilia, B. Braun AG, Melsungen, Germany) to a concentration of 1 mg/ml and further diluted with DMEM 2× (Invitrogen Corp., Karlsruhe, Germany) containing 20% FBS and 2% P/S to a concentration of 0,5 mg/ml. The pH was equilibrated to 7,0 with NaOH. Afterwards the fibrinogen-cell-solution and the collagen solution were mixed 1:1 and pipetted into 24-well-plates (Corning Incorporated, NY, USA) and 6 IU thrombin per ml fibrinogen (Tisseel-kit, Baxter AG, Vienna, Austria,) were added to the wells. Each specimen had a volume of 0,7 ml (height: approximately 0,8 cm, diameter: 1,5 cm), collagen-concentration of 0,25 mg/ml, fibrin-concentration of either 5,0 mg/ml or 2,5 mg/ml (table 1) and 731 KU aprotinin per specimen. The collagen-fibrin clots were totally gelled after 30 minutes and were then covered with growth medium. After the second day, growth medium was replaced with DMEM containing 2% donor horse serum (DHS, Biochrom AG, Berlin, Germany) and 1% P/S.

### Cell viability/TUNEL-assay

For this experiment, collagen-I fibrin gels were composed as in group 4 (table 1). However, instead of aprotinin, genipin solution (Wako Chemicals GmbH, Neuss, Germany) at a concentration of 10 mg/ml was added in quantities of 10, 21, 56, 100 μl to the resuspended cells. The clots as well as collagen sponges were seeded with either 100.000 or 500.000 cells and harvested after 4 days in culture. Paraffin sections were taken from two defined regions of each of the specimens, one peripheral and one central section.

Sections were stained using the fluorescein DNA fragmentation detection kit (FragEL kit, Calbiochem/Merck Biosciences, Darmstadt, Germany) according to the manufacturer's instructions, digitally photographed and evaluated by ImageJ-program. Apoptosis rate was calculated as the ratio of apoptotic cells to total cell number. All groups were tested against the control group (group 4, containing aprotinin) and the two-tailed student's t-test was used for statistical analysis. Results were considered significant when p-value was less than 0,05.

### Immunohistochemistry

Cryosections were performed after 1, 2, 3, 4, 5 and 7 days and stained for two muscle-specific markers – MyoD and desmin – to assess cell differentiation and DAPI (Diamidine-phenylindole-dihydrochloride, Applied Science/Roche, Indianapolis; IN, USA) as nucleic counterstain. First, sections were fixed in methanol, blocked with 5% FBS/0,25% TritonX 100 (Sigma Aldrich, St. Louis, MO, USA) in PBS (PBS-Dulbecco 1×, Biochrom AG, Berlin, Germany) for 30 minutes and rinsed with PBS. Then sections were covered with the primary antibody for desmin (monoclonal mouse IgG, Dako Cytomation, Carpinteria, CA, USA) or MyoD (polyclonal rabbit IgG, Santa Cruz Biotechnology, CA, USA) and incubated for 2 hours. After rinsing the slides with PBS, the secondary antibody against mouse (goat anti-mouse, cy-2-conjugated, Jackson ImmunoResearch, Suffolk, UK) and rabbit respectively (goat anti-rabbit, cy-3-conjugated) were added and incubated for 1 hour. Finally, the specimens were counterstained with DAPI for 30 minutes and slides were covered with mounting media (Fluoprep, Biomérieux, Marcy l'Etoile, France). The slides were evaluated and digitally photographed with fluorescence microscopy (Leitz DMRBE and camera, Leica Microsystems, Wetzlar, Germany).

### Cell proliferation

To measure the cell proliferation rate, specimens of every group were harvested on day 1, 2, 3, 4, 5, 7 and 14 and embedded in paraffin. From every specimen four sections were done in a standardized fashion: two from the peripheral (10–100 μm to the surface) and two from the central part of the 3D-construct. Sections were subsequently stained with DAPI and four images were recorded of every slide. The number of DAPI-stained cells was counted semi-automatically with ImageJ program. For statistical analysis parametric one way ANOVA with repeated measurements was used with the Tukey post hoc test for multiple comparisons.

### Collagen sponge

Collagen sponges were provided by BD Biosciences (Collagen Composite, Bedford, MA, USA) and were composed of bovine collagens type I and III. Physical characteristics of the collagen sponges: 3 mm height, diameter of 5 mm, average pore size: 100–200 μm. The sponges were placed into 24-well plates and incubated in growth medium for 45 minutes at 37°C. Sponges were seeded with either 100.000 cells or 500.000 cells by pipetting the resuspended cells directly onto the matrix which was then covered with growth medium. After 24 hours the sponges were transferred into new wells to prevent cells adherent to the bottom of the wells from growing into the matrix. Further treatment was the same as described for the fibrin-collagen gels. The collagen sponges were harvested after 7 and 14 days in culture. Immunochemistry for MyoD and desmin and DAPI staining for statistical evaluation of the cell proliferation rate as well as the assessment of apoptosis rates after 4 days by TUNEL-assay were performed as described for the fibrin-collagen gels. Additionally, the collagen sponges were evaluated by scanning electron microscopy (Quanta 200, FEI Company, Hillsboro, USA). Therefore, sponges were first fixed with paraformaldehyd then dehydrated in acetone and finally dried with the critical point method, using liquid CO_2 _as exchanging medium (CPD 030, Bal-Tec AG, Liechtenstein). Prior to SEM-analysis the samples were sputtered with gold (SCD 040, Bal-Tec AG, Liechtenstein).

### OPLA^® ^scaffolds

OPLA^® ^scaffolds were also provided by BD Biosciences (Collagen Composite, Bedford, MA, USA). The scaffold was synthesized from D, D, L, L-polylactic acid. Physical dimensions of the scaffold: 4 mm high, 4,5 mm in diameter. Approximately 100–200 μm was quoted as the average pore size of the OPLA scaffolds. Modulus of elasticity was assessed by the supplier and valued 0,92 ± 0,03 MPa.

Seeding procedure and cultivation of the OPLA^® ^scaffolds and collagen sponges were identical. However, OPLA^® ^dissolved completely in ethanol and acetone and fixation for SEM and paraffin sections was therefore not possible. Thus, our analysis of OPLA^® ^was limited to SEM of dry OPLA^® ^scaffolds for evaluation of surface property, immunocytochemistry of cryosections and analysis of apoptosis rate.

### Electrospinning of collagen nanofibres and cell seeding

The electrospinning process was performed as previously described [10]. Collagen nanofibres were electrospun parallel aligned or without alignment as control. Bovine collagen type I (Sigma-Aldrich, Taufkirchen, Germany) was dissolved in hexafluoroisopropanol (Sigma-Aldrich, Taufkirchen, Germany) at a concentration of 5% w/v (needle bore size: 0,6 mm, distance between needle tip and counter electrode: 12 cm). A voltage of 12 kV was applied and the nanofibers were then deposited as nonwovens on glass plates (16 mm diameter) fixed on the counter electrode. For orientated nanofibres, the glass plates were fixed to a roll rotating at 3500 rpm. The samples were then incubated for 1 h in vacuum at room temperature and afterwards heated slowly to 110°C for 120 h. The samples were cooled down to room temperature overnight and framed with a brim of PLLA (Poly-L-lactid, 4% wt, Boehringer Ingelheim Pharma GmbH, Ingelheim, Germany) to prevent the nanofibers from floating off the glass plates. At last the samples were plasma-sterilized with H_2_O_2_.

Shortly before cell seeding, the collagen nanofibers were soaked in DMEM for 30 minutes. The scaffolds were placed in 12-well plates and 100 μl growth medium containing 20.000 myoblasts in passage 3 were then pipetted directly onto each scaffold. After incubation at 37°C for 2h the wells were filled with 2ml growth medium. The scaffolds were transferred to new wells 24h after seeding. Cultivating conditions were equal to the other matrices.

Nanofibre constructs were evaluated by phase contrast microscopy (Fluovert FU, Leica Microsystems, Wetzlar, Germany), immunochemistry and scanning electron microscopy as described above.

### RNA isolation, cDNA synthesis and Light-Cycler-PCR

In the four groups of collagen-I-fibrin gels the expression rates of desmin, MyoD and MEF-2d (table 2) were analyzed. Gene expression in other matrices were not analyzed since the differences in mechanical and chemical properties of gel matrices, sponges and two-dimensional matrices are expected to be too great to control for a sensible quantitative PCR setting. Total RNA isolated from skeletal muscle tissue of newborn Lewis rats was used as calibrator because neonatal muscle tissue, in contrast to adult muscle tissue, is known to present a high expression of the muscle specific markers desmin, MoyD and MEF-2d and GAPDH was chosen as endogenous control. For every group, five scaffolds were harvested after 14 days in culture, frozen in liquid nitrogen and ground with mortar and pestle. RNA was then isolated via QiaShredder and Micro-RNeasy-kit (Qiagen GmbH, Hilden, Germany) according to the manufacturer's instructions. Concentration and purity of the pooled RNA was assessed for every group photometrically by 260/230-ratio and 260/280-ratio using an Eppendorf Biophotometer (Eppendorf AG, Hamburg, Germany). Only mRNA with sufficient 260/280 and 260/230-ratio was reverse-transcribed with Sensiscript^®^-RT-kit and oligo-dT primers for cDNA synthesis (Qiagen GmbH, Hilden, Germany). For quantitative PCR with Light Cycler (Bio-Rad iCycler iQ5, Bio-Rad Inc., Hercules, CA, USA) we used SYBR GreenER kit (SYBR GreenER qPCR SuperMix, Invitrogen Corp., Karlsruhe, Germany) according to the distributor's instructions. Samples were tested as dublicates whereas deviations of 1,5 threshold cycles were tolerated. For data evaluation the ΔΔC_T_-method was used and only threshold cycles before cycle 35 were defined as valid.

**Table 2:** Expression rates of desmin, MyoD and MEF-2d

Primer qPCR	Desmin	MyoD	MEF-2	GAPDH
Fwd	5'-ata ccg aca cca gat cca gtc c-3'	5'-aga ggg aag gga aga gca gaa g-3'	5'-tgc tgc tct cac tgt cac tac-3'	5'-caa cga ccc ctt cat tga cc-3'

Rev	5'-tcc ctc atc tgc ctc atc aag g-3'	5'-gca gca gca aca aca acc ag-3'	5'-ttc acg act tgg gga cac tg-3'	5'-ttc tca gcc ttg act gtg cc-3'

## Authors' contributions

JPB designed and supervised the study, drafted the manuscript and participated in all stages of the work. DK carried out the experimental work and wrote the manuscript. OB drafted and corrected the manuscript. MR, RD and JHW contributed the material for the nanofiber scaffold and carried out the electrospinning process. AA, UK and REH provided materials and analysis tools and contributed to the conception of the study. EP performed the statistical analysis. All authors read and approved the final manuscript.
